# Seasonal plasticity in morphology and metabolism differs between migratory North American and resident Costa Rican monarch butterflies

**DOI:** 10.1002/ece3.9796

**Published:** 2023-02-22

**Authors:** Ayşe Tenger‐Trolander, Cole R. Julick, Wei Lu, Delbert André Green, Kristi L. Montooth, Marcus R. Kronforst

**Affiliations:** ^1^ Department of Ecology and Evolution University of Chicago Chicago Illinois USA; ^2^ Ecology and Evolutionary Biology University of Michigan Ann Arbor Michigan USA; ^3^ School of Biological Sciences University of Nebraska‐Lincoln Lincoln Nebraska USA

**Keywords:** *Danaus plexippus*, metabolic rate, migration phenotypes, monarch butterfly, seasonal plasticity, wing morphology

## Abstract

Environmental heterogeneity in temperate latitudes is expected to maintain seasonally plastic life‐history strategies that include the tuning of morphologies and metabolism that support overwintering. For species that have expanded their ranges into tropical latitudes, it is unclear the extent to which the capacity for plasticity will be maintained or will erode with disuse. The migratory generations of the North American (NA) monarch butterfly *Danaus plexippus* lead distinctly different lives from their summer generation NA parents and their tropical descendants living in Costa Rica (CR). NA migratory monarchs postpone reproduction, travel thousands of kilometers south to overwinter in Mexico, and subsist on little food for months. Whether recently dispersed populations of monarchs such as those in Costa Rica, which are no longer subject to selection imposed by migration, retain ancestral seasonal plasticity is unclear. To investigate the differences in seasonal plasticity, we reared the NA and CR monarchs in summer and autumn in Illinois, USA, and measured the seasonal reaction norms for aspects of morphology and metabolism related to flight. NA monarchs were seasonally plastic in forewing and thorax size, increasing wing area and thorax to body mass ratio in autumn. While CR monarchs increased thorax mass in autumn, they did not increase the area of the forewing. NA monarchs maintained similar resting and maximal flight metabolic rates across seasons. However, CR monarchs had elevated metabolic rates in autumn. Our findings suggest that the recent expansion of monarchs into habitats that support year‐round breeding may be accompanied by (1) the loss of some aspects of morphological plasticity as well as (2) the underlying physiological mechanisms that maintain metabolic homeostasis in the face of temperature heterogeneity.

## Graphical abstract

Environmental heterogeneity in temperate latitudes is expected to maintain seasonally plastic life‐history strategies that include the tuning of morphologies and metabolism that support overwintering. Whether recently dispersed populations of monarch butterflies, such as those living in Costa Rica, retain ancestral seasonal plasticity is unclear. We investigated the differences in seasonal plasticity by rearing North American and Costa Rican monarchs in summer and autumn in Illinois, USA, and measured the seasonal reaction norms for aspects of morphology and metabolism related to flight.

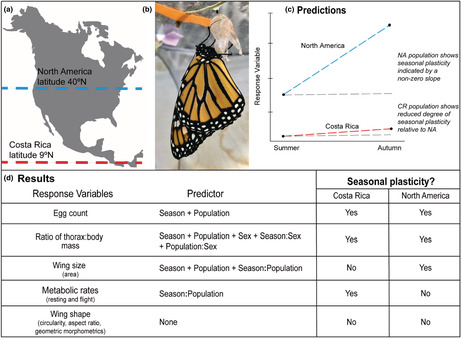

## 1 INTRODUCTION

Fluctuating seasonal environments in temperate habitats are expected to favor the evolution of overwintering strategies that are, by their nature, plastic responses to the environment (Kingsolver & Huey, 1998; Moran, 1992). For some species, these strategies involve changes in physiology and morphology that accompany overwintering in place, while for other species, they involve physiological and morphological changes that support seasonal migration (Arnold et al., 2004; Butler & Woakes, 2001). Theory for the evolutionary maintenance and loss of plasticity has been well developed (de Jong, 1990; Gavrilets & Scheiner, 1993; Gomulkiewicz & Kirkpatrick, 1992; Moran, 1992; Van Tienderen, 1991; Via & Lande, 1985, 1987), and there are established empirical frameworks and organismal systems for investigating the evolutionary dynamics of seasonal plasticity (Kingsolver & Huey, 1998; Scheiner, 1993). Trait plasticity may be lost when species ranges expand out of temperate, seasonal environments, and into tropical, constant environments. This loss may occur via costs of plasticity that include a reduced efficacy of selection (DeWitt et al., 1998; Kawecki, 1994; Van Dyken & Wade, 2010; Van Tienderen, 1991; Whitlock, 1996) or genetic assimilation whereby adaptation to a new constant environment fixes the trait value (Lande, 2009; Price et al., 2003; Schleicherová et al., 2013; Waddington, 1961; Wan et al., 2018).

Trait plasticity may be a key factor enabling population persistence as environments across the globe become more variable (Catullo et al., 2019; Matesanz & Ramírez‐Valiente, 2019; O'Connor et al., 2012; Price et al., 2003; Sgrò et al., 2016), motivating a better empirical understanding of how and when plasticity is lost. The loss of plasticity through assimilation, where some populations evolve a fixed phenotype that maximizes fitness in a new, stable environment (Aubret & Shine, 2009; Corl et al., 2018), has been demonstrated in a handful of systems, including at a mechanistic and genetic level for wing coloration in common buckeye butterflies (van der Burg et al., 2020). A series of studies by Cooper et al. (2012, 2014) suggest that cellular membrane plasticity in phospholipid composition can be eroded through disuse or via costs of maintaining plasticity in fruit fly populations evolved in constant thermal environments. Relaxed selection at cold‐acclimation genes coincides with range expansion into warmer latitudes in *Arabidopsis* (Zhen et al., 2011; Zhen & Ungerer, 2008). However, seasonal plasticity is complex in that it involves suites of traits to support divergent physiologies or life histories across seasons (Williams et al., 2017; Wilsterman et al., 2021). Investigating how this complex multi‐trait plasticity is lost (e.g., piecemeal vs wholesale loss) when species ranges expand into less seasonal latitudes may provide insight into both the mechanisms of trait integration and trait loss, as well as identify the aspects of seasonal plasticity that may be retained and respond to different environmental cues in the new environment.

North American (NA) monarch butterflies (*Danaus plexippus*) are well known for their long‐distance seasonal migration plasticity that meets different dispersal, reproductive, and energetic demands across generations (Reppert & de Roode, 2018). The migratory state is induced between late summer and early fall by decreasing photoperiod, cooler, and fluctuating temperatures and senescing host plants (Goehring & Oberhauser, 2002). Summer generations are short‐lived and reproduce shortly after adult eclosion. The autumn/winter generations live for 8–12 months during which they migrate to their overwintering grounds in Mexico where they remain in reproductive diapause until the following spring. They then migrate back into the Southern United States and successive generations recolonize northern latitudes. In North America, seasonally variable environmental conditions are predicted to maintain plasticity for many aspects of morphology and physiology that support the different life‐history strategies in summer versus autumn/winter generations. Notably, previous studies comparing summer (non‐migratory) and autumn (migratory) generation monarchs have shown that NA monarchs eclose in reproductive diapause, have increased longevity and cold tolerance, have greater fat stores, have differences in sun compass neuropil volume, and have a strong drive to fly south in autumn compared with summer‐eclosing monarchs (Barker & Herman, 1976; Brower et al., 2006; Goehring & Oberhauser, 2002; Heinze et al., 2013; Herman & Tatar, 2001; Tenger‐Trolander et al., 2019; Zhu et al., 2008, 2009).

North American monarchs have expanded their range through multiple independent dispersal events into tropical latitudes that lack seasonal heterogeneity and support resident, year‐round breeding populations (Zhan et al., 2014), making monarchs a good system to investigate the loss of complex multi‐trait plasticity. These populations are descendants of the migratory NA population, but no longer migrate and differ in two migration‐relevant phenotypes – wing size and sun compass neuron tuning to sunlight (Altizer & Davis, 2010; Freedman et al., 2020; Nguyen et al., 2021). Today, non‐migratory populations can be found in Central and South America, the Caribbean, the Iberian Peninsula, Morocco, the Pacific Islands, Australia, and New Zealand (Pfeiler et al., 2017; Zhan et al., 2014). In Australia, there are both migratory and non‐migratory populations that exhibit plasticity in reproductive development (Dingle et al., 1999; Freedman et al., 2018; James, 1984), suggesting that some aspects of seasonal migration plasticity may be maintained within some dispersed populations.

**FIGURE 1:**
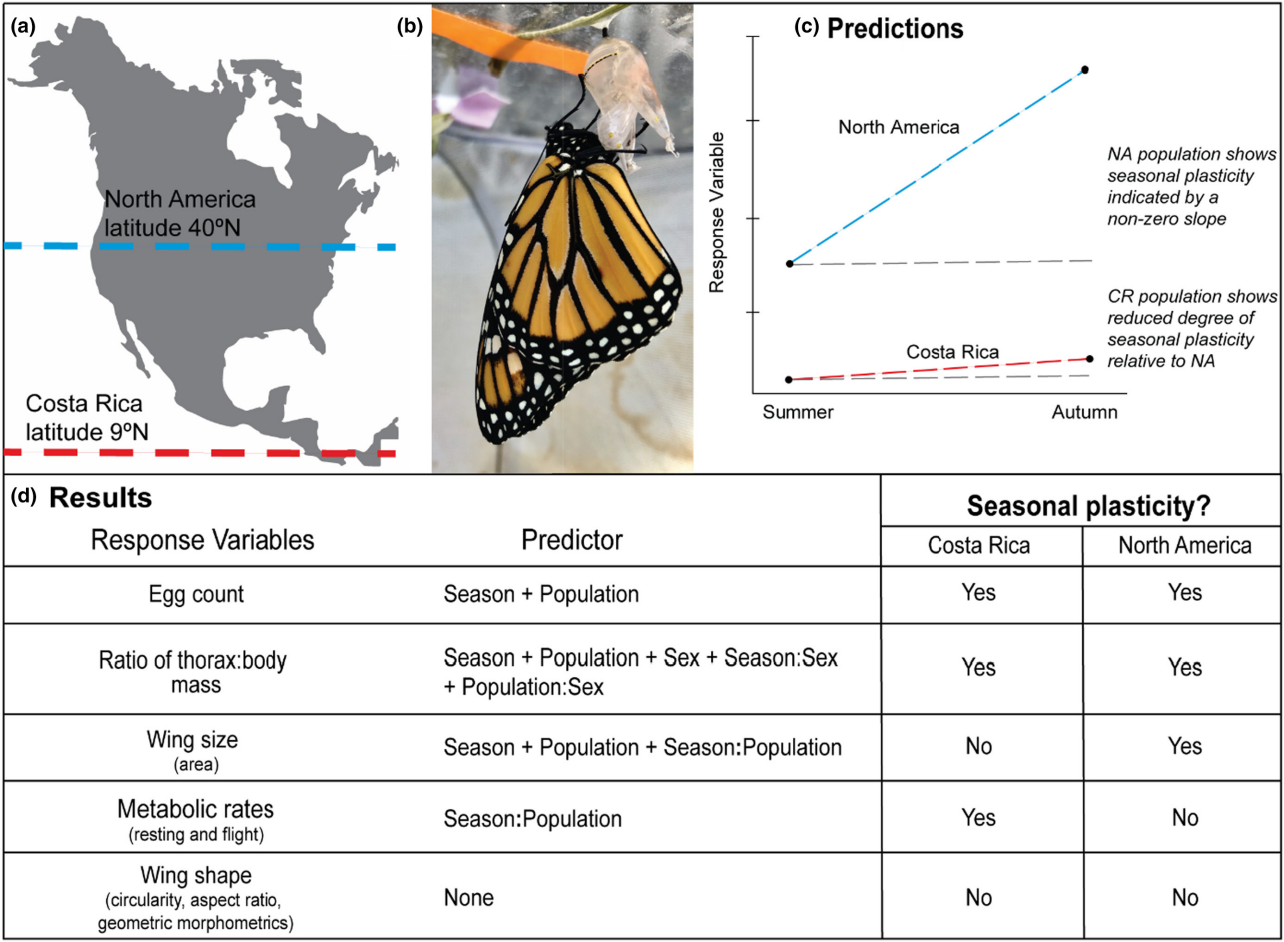
Summary of the experiment, predictions, and main findings investigating seasonal plasticity in Costa Rican (CR) and North American (NA) monarchs. (a) Map of North and Central America indicating NA (blue) and CR (red) monarch respective latitudes. (b) Photo of a newly eclosed monarch butterfly. (c) Prediction and potential outcomes of a possible response variable (i.e., a trait). Populations may differ in trait value regardless of seasonal rearing condition (i.e., a difference in y‐intercept between populations). Populations may also differ in seasonal trait plasticity. A non‐zero reaction norm between summer and autumn trait values within a population indicates the presence of seasonal plasticity (blue and red vs gray lines), and differences in reaction norm between populations indicate differences in degree of plasticity (different slope of blue and red line). (d) List of traits measured in this study, the independent variables (population, rearing season, and sex) that explained significant variance in the trait, and whether each population exhibited seasonal trait plasticity.

Here, we quantified plasticity across summer‐ and autumn‐reared generations of NA monarchs for wing morphology and metabolic traits that are thought to be related to long‐distance migration, and then we tested whether Costa Rican (CR) monarch butterflies have lost or decreased seasonal plasticity in these traits (Figure 1). We compared NA and CR monarchs for traits known or expected to affect migratory success in NA monarchs; however, we noted that these traits do not in themselves determine whether a monarch population is migratory. CR monarchs are generally considered non‐migratory by monarch researchers for two reasons. They are found in Costa Rica year round (Altizer & Davis, 2010; Haber, 1993; Haber & Stevenson, 2004; Pfeiler et al., 2017; Zhan et al., 2014), and relative to the distance of the NA monarch seasonal migration, CR monarchs only disperse short distances when their host plant (*Asclepias curassavica*) becomes unavailable (Haber, 1993; Haber & Stevenson, 2004). We hypothesized that the lack of temperate‐latitude seasonal selection over many generations since their dispersal and isolation from NA monarchs has resulted in changes to plasticity in migration‐relevant traits in CR monarchs because the long‐distance migration is no longer part of their life history. Using a common garden experiment with outdoor seasonal rearing of NA and CR monarchs, we tested the prediction that NA migratory populations have greater plasticity in response to seasonal rearing conditions than do CR non‐migratory populations that no longer experience temperate‐latitude seasonality in temperature, day length, and host‐plant availability (Figure 1).

## 2 METHODS

### 2.1 Trait selection

We selected the physiological and morphological traits that are likely subject to different selective pressures depending on seasonal environmental heterogeneity, including egg count, thorax and abdomen mass, wing size, wing shape, and metabolic rate. Plasticity in these traits is thought to impact monarch success in reproduction, migration, and overwintering. We counted the number of mature oocytes as a measure of reproductive arrest, a well‐known phenomenon in migrating NA monarchs that is correlated with their longevity and overwintering strategy (Barker & Herman, 1976; Goehring & Oberhauser, 2002). We measured the resting and maximal flight metabolic rates to quantify plasticity in energy demand that supports adult maintenance and flight. We massed the thorax and abdomen separately to estimate mass associated with the flight muscles and with the reproductive organs and fat body, respectively. We measured the forewing size and shape as traits associated with flight efficiency. Larger wings generate more lift due to lower wing loading, and more narrow wings with high aspect ratios decrease drag (Dudley, 2000; Egbert & Belthoff, 2003; Lockwood et al., 1998; Senar et al., 1994; Swaddle & Witter, 1998; Wang, 2004; Winkler & Leisler, 1992).

### 2.2 Seasonal rearing

We reared two generations (summer and autumn) of NA and CR monarch butterflies outdoors in Chicago, IL, in 2016 and 2017. Rearing was done under permits from USDA‐APHIS. Butterflies that emerged in July and August were designated as the summer generation, and those that emerged in September and October were designated as the autumn generation. In both years, the autumn generations were the offspring of the summer generation. In 2016, we measured the metabolic traits in female and male adult monarchs and counted the number of mature oocytes in the females. We repeated these measurements for monarchs reared in 2017, with the addition of morphological measurements, including body mass, forewing size, and forewing shape.

### 2.3 Sample sizes

We reared 576 monarchs (149 individuals in 2016 and 427 individuals in 2017) and measured 573 of these for at least one trait. We measured 179 individual's metabolic rates, dissected 165 females to count the number of mature oocytes present in the abdomen, dried and massed 184 individuals, assayed the geometric morphometric shape traits of 254 individuals, and measured the forewing size and shape traits for 237 individuals. Some individuals were used for multiple measurements, but those for which we counted oocytes could not be used for mass measurements and vice versa. In addition, butterflies for which we measured metabolic traits were more likely to be tattered and excluded from wing trait measurements. Further details on the number of individuals measured for each trait by rearing year, season of development, and population can be found in Table 1.

**TABLE 1:** Sample sizes for all traits measured.

	Sample size in 2016 (*N* = 149)			
	North America (*N* = 85)		Costa Rica (*N* = 64)	
	Summer (*N* = 53)	Autumn (*N* = 32)	Summer (*N* = 44)	Autumn (*N* = 20)
Phenotypes				
Metabolic rate (MR)	36	32	25	12
Mature oocyte (MO)	33	10	32	11
MR + MO	16	10	13	3

	Sample size in 2017 (*N* = 427)			
	North America (*N* = 233)		Costa Rica (*N* = 194)	
	Summer (*N* = 159)	Autumn (*N* = 74)	Summer (*N* = 114)	Autumn (*N* = 80)
Phenotypes				
Metabolic rate (MR)	20	7	30	17
Mature oocyte (MO)	33	20	5	16
MR + MO	4	0	0	4
Mass	55	36	60	33
Wings	83	61	48	62
Mass + wings	7	32	18	32

*Note*: Top: For samples reared in 2016, we assayed metabolic rate (MR) and number of mature oocytes (MO). MR + MO indicates the subset of individuals from the MR and MO row tallies for which we have measurements of both traits for the same individual. Bottom: For samples reared in 2017, in addition to metabolic rate assays and oocyte counts, we measured the morphological traits using wings and bodies. MR + MO indicates the subset of individuals from the MR and MO row tallies for which we have measurements of both traits for the same individual, and Mass + Wings indicates the subset of individuals from the Mass and Wings row tallies for which we have measurements of both traits for the same individual.

### 2.4 Genetic composition

We derived CR monarchs from ~20 pupae obtained from El Bosque Nuevo butterfly farm in Costa Rica in 2016 and again in 2017. The breeder maintains outdoor greenhouses where adults mate freely and regularly supplements the population with wild‐caught monarchs from the surrounding area. While Central and South American monarchs remain the most genetically similar to NA monarchs, perhaps as a result of continuing gene flow (Freedman et al., 2020; Pierce et al., 2014), their estimated divergence time of 2000–3000 years from the North American population is the largest among the three dispersals (Zhan et al., 2014).

All NA monarchs reared in 2017 were derived from wild‐caught NA monarchs captured in Chicago, IL, and morphological traits were only measured in 2017 (Table 1). The NA monarchs reared in 2016 were derived from two sources: wild‐caught and commercially sourced NA individuals. At the time of the first common garden experiment, we were not aware of the genetic distinctiveness of the commercial NA lineage compared with the wild NA population (Tenger‐Trolander et al., 2019). Of the 179 individuals assayed for MR (Table 2), only 18 individuals were purely commercial (15 summer‐reared and 3 autumn‐reared), 21 were NA/Commercial F1 crosses reared in summer, and 29 were backcrosses (NA/Commercial F1 backcrossed to NA) reared in autumn. While a proportion of pure commercial NA monarchs have lost the propensity to orient south in response to autumn rearing conditions (Tenger‐Trolander et al., 2019; Tenger‐Trolander & Kronforst, 2020), the backcrosses (NA/Commercial F1 backcrossed to NA) reared in autumn showed clear southern orientation (Tenger‐Trolander et al., 2019). The commercial lineage also enters reproductive diapause when reared outdoors in autumn implying that some physiological responses in the wild and commercial NA populations are similar (Tenger‐Trolander et al., 2019).

**TABLE 2:** Combined sample sizes for metabolic rate measurements of monarchs reared in 2016 and 2017.

	Sample sizes 2016 ± 2017 (*N* = 179)			
	Summer (*N* = 111)		Autumn (*N* = 68)	
	Females	Males	Females	Males
North American	29	27	16	23
Costa Rican	32	23	14	15

### 2.5 Animal husbandry

We housed the monarchs from their respective populations in medium size (91.5 cm × 30.5 cm^2^) mesh pop‐up cages outdoors with access to the host plant, *Asclepias syriaca*. After females laid eggs, we transferred the eggs to small (30.5 cm^3^) outdoor mesh pop‐up cages and fed larvae on a diet of wild‐collected *A. syriaca* cuttings. All pop‐up cages were contained inside two large outdoor 1.83 m^3^ mesh cages separated by population of origin. As individuals eclosed, they were collected as virgin males and females, labeled with a unique ID, and left outdoors for a minimum of 3 days. Adults were then either shipped to Lincoln, NE, for metabolic measurement or measured for morphological traits in Chicago. Adult butterflies were shipped overnight in glassine envelopes, spending between 12 and 24 h in a dark cardboard box. Upon arrival, butterflies were separated by sex and housed in large collapsible butterfly cages in a laboratory space with natural light. Prior to metabolic measurements, all individuals were given at least 48 h to acclimate. Individuals kept in Chicago were also separated by sex and housed outdoors in Chicago, IL, until frozen for morphological trait measurements. All butterflies had access to a constant supply of artificial nectar (Birds Choice Butterfly Nectar, Chilton, WI).

In 2017, summer‐reared monarchs were shipped back to Chicago from Lincoln to found the autumn generation due to a summer die‐off caused by the spillover of the pesticide permethrin from a neighboring yard in Chicago. None of the summer‐reared monarchs that we measured were present when this exposure occurred, and the subsequent autumn generation of monarchs was founded by individuals not present during the die‐off.

### 2.6 Mature oocyte counts

Females were kept separately from males and never mated. We dissected females by making a longitudinal cut down the abdomen to remove eggs. We then counted the number of mature oocytes present. Immature and mature oocytes in monarchs are distinguished by the shape of the chorion. A smooth chorion surface indicates an immature oocyte, while a chorion with ridges is considered mature.

### 2.7 Body mass measurements

We removed the wings, antennae, head, and legs from the body. We separated the thorax and abdomen and dried them at 60°C in an incubator with a silica crystal desiccant for 72 h. After drying, we weighed the thorax and abdomen both separately and together on an analytical balance.

### 2.8 Wing size and shape measurements

**FIGURE 2:**
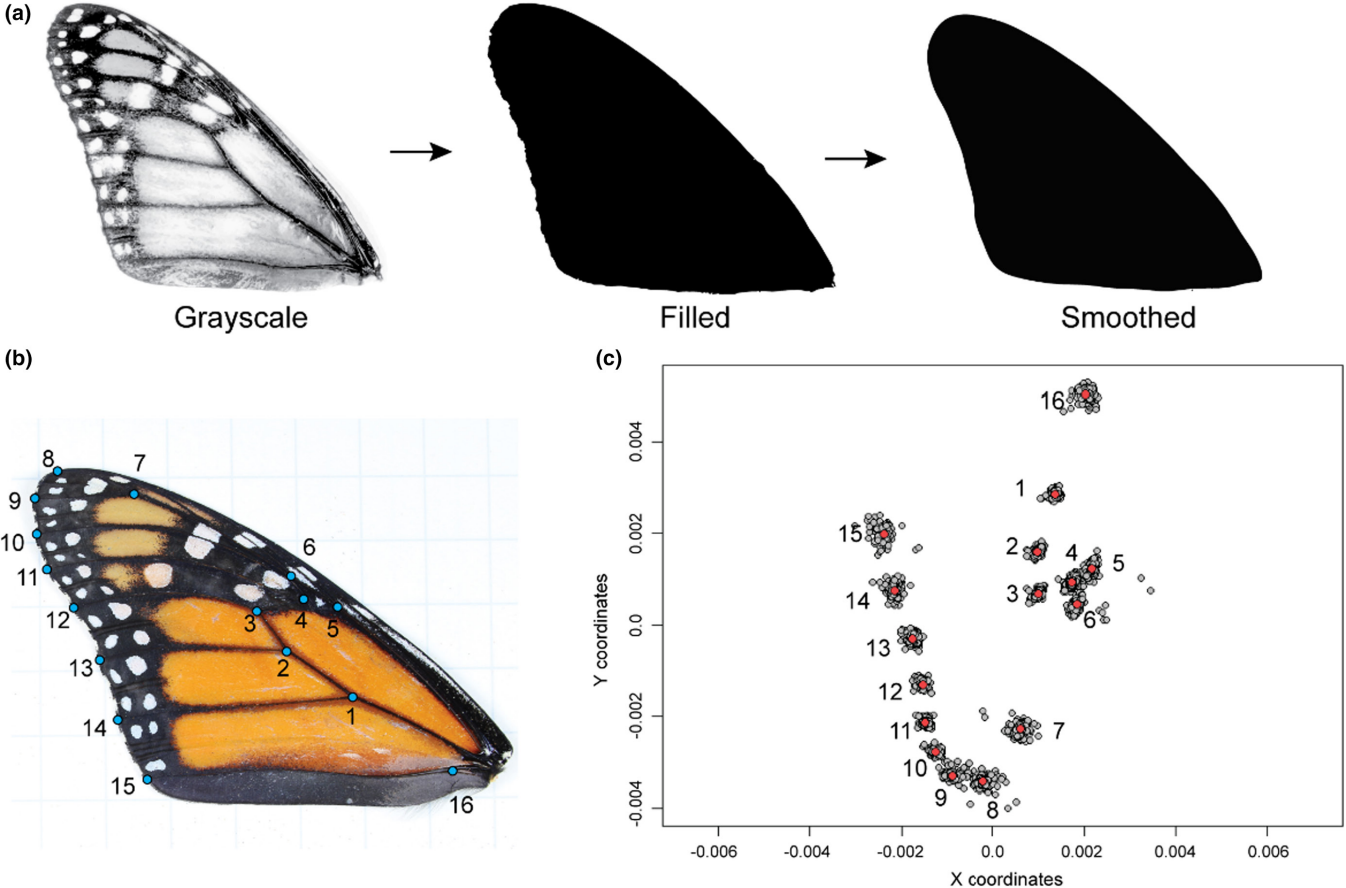
(a) Example of a monarch forewing being processed in ImageJ. The photo is converted to an 8‐bit black/white image. The non‐black pixels within the forewing are filled with black. Once filled, the edges of the wing are smoothed using the “Shape smoothing” plugin. (b) Blue dots indicate the positions of 16 landmarks on monarch butterfly forewing. (b) Plot of all specimens' landmarks after general Procrustes alignment in gray. (c) Red dots are the consensus mean coordinates for each landmark. Landmarks numbered 1 through 16 correspond to the same vein intersections and ends seen in section B of this figure.

We placed a single forewing and hindwing on a sheet of gridded paper with 0.635 cm squares or on a white sheet of paper with a metric ruler in view. We photographed the wings using a DSLR Canon EOS 70d camera with an 18–55 mm lens. We scaled each photo by number of pixels/cm and converted the color photos to 8‐bit black/white images in ImageJ (Rueden et al., 2017; Schindelin et al., 2012). We filled in non‐black portions of the forewing with black to measure area (Figure 2a). Before measuring area or shape attributes, we smoothed the contours of the forewings with the ImageJ plugin “Shape smoothing” (Erdenetsogt & Wagner, 2016). “Shape smoothing” applies a Fourier transformation to gain Fourier descriptors (FDs). We kept 0.35% of FDs relative to the total number of FDs identified in the image (Figure 2a). We then measured the area (in cm^2^), aspect ratio (length/width), and circularity (4π*area/perimeter^2^) of each forewing in ImageJ (Rueden et al., 2017). To measure aspect ratio, ImageJ finds the longest length (major axis) and width (minor axis) of the object while maintaining the perpendicular intersection of both lines and divides the major axis length by the minor. Higher circularity scores indicate a more circular wing shape, whereas lower scores indicate more polygonal or angular shapes. Circularity is different than roundness (4*area/(π*major_axis^2^)). For example, a hexagon has high circularity and low roundness, whereas an oval has low circularity and high roundness.

We also used 2D landmark‐based geometric morphometrics to assess the shape differences. Using the software tpsDIG2ws, we placed 16 landmarks at homologous points (vein intersections and margins) on each forewing (Rohlf, 2006) (Figure 2b). We analyzed the resulting landmark data in R using the package “Geomorph” (Adams & Collyer, 2020). We performed a general Procrustes analysis that removed differences in orientation and size, allowing us to focus exclusively on shape differences (Figure 2c). We then calculated the mean shape which is the average landmark coordinates for a set of aligned wings (Figure 2c, red dots). For each of the 16 landmarks, we calculated the distance between the individual's coordinates and the group mean coordinates and summed those distances to find each specimen's total distance from the mean shape.

### 2.9 Metabolic rate measures

Using flow‐through respirometry, we estimated the resting or routine metabolic rate (MR) and the maximal flight MR from the volume of CO_2_ (VCO_2_) produced by individual adult monarchs ranging from 3 to 45 days old. While not ideal, this age range was a consequence of shipping logistics between Chicago and Lincoln. Age was not a significant predictor of MR, even when controlling for mass in an analysis of covariance (routine MR, *p* = .157; flight MR, *p* = .310). Older butterflies tended to be smaller (effect of age on mass, *p* = .009), and variation in mass was accounted for in our statistical analysis of MR (see below in *Statistical Analyses*). Butterflies were placed in a 3.3‐L glass cylindrical container covered with a piece of black velvet cloth ensuring a complete darkness during resting MR measurements. CO_2_‐free, dry air was pumped through the container at a rate of 3 L/min using a dual pump system (Sable Systems International, Las Vegas, NV, USA) coupled with a mass‐flow valve (Sierra Instruments, Monterey, CA, USA). After the air left the measurement container, it was subsampled at 100 mL/min using a SS‐4 Sub‐Sampler pump (Sable Systems International, Las Vegas, NV, USA), scrubbed of water, and then passed into a high‐performance CO_2_/H_2_O differential gas analyzer (LI‐7000, Li‐Cor, Lincoln, NE, USA) to quantify CO_2_. All MR data were collected using the Expedata software package (Sable Systems International, Las Vegas, NV, USA).

Individuals rested in the cloth‐covered chamber at 21°C for a minimum of 25 min prior to metabolic rate measurement. Before removing the cloth, we recorded resting MR until a stable resting MR was established. We then removed the cloth and exposed the individual to full‐spectrum UV light. After 30 s of light exposure, we induced flight by gently shaking the container. We recorded 10 min of CO_2_ production during flight. If butterflies stopped flying during this 10‐min period, we gently shook the chamber to induce flight. After flying for 10 min, we turned off the light and covered the container to allow the butterfly to return to a stable resting MR. While 21°C is cooler than others' have used to measure flight metabolic rate (e.g., Pocius et al., 2022; Zhan et al., 2014), we chose this because it was the common garden temperature at which all monarchs were being held in the lab prior to measurements. We experienced no issues inducing flight, which was likely facilitated by warming due to the full‐spectrum UV light and monarch thermoregulatory behavior (Masters et al., 1988). The maximal rate of CO_2_ production sustained over a 1‐min period during the 10‐min flight was used as our estimate of maximal flight MR. This measure of maximal flight metabolic rate is not meant to approximate metabolic rate during migratory flight but rather is a proxy for maximal metabolic rate during activity. Before and after each metabolic measurement, baseline CO_2_ values were recorded and drift‐corrected using the two‐endpoint method in Expedata. To compensate for a response lag in the respirometry system, we utilized the “Z‐transformation” function (instantaneous transformation) in Expedata. Raw CO_2_ values were converted from parts per million to mL/h.

### 2.10 Statistical analyses

For analyses of morphological traits, we used the R package “glmulti” to automatically select the best fit generalized linear model for each trait and determine which of the independent variables (sex, population, season) were significant predictors of the measurements (Calcagno & de Mazancourt, 2010; R Core Team, 2013). We fit thorax mass (grams), thorax: body mass ratio, forewing area (cm^2^), and abdomen mass (log‐transformed) within the Gaussian family as these traits were normally distributed (Table 3). For egg counts, we fit the model with a negative binomial distribution (Table 4).

**TABLE 3:** Candidate general linear models for traits: abdomen mass, thorax mass, thorax: body mass ratio, and forewing area.

	BIC	Weight
**Abdomen mass candidate models**		
Abdomen mass ~ 1 ± Season + Sex + Sex:Season	104.784	0.6458
Abdomen mass ~ 1	107.794	0.1434
Abdomen mass ~ 1 ± Season	109.598	0.0582
Abdomen mass ~ 1 ± Sex	109.858	0.0511
Abdomen mass ~ 1 ± Season + Sex + Population ± Sex:Season	109.994	0.0477
Abdomen mass ~ 1 ± Season + Sex	112.140	0.0163
Abdomen mass ~ 1 ± Season + Sex + Population ± Sex:Season + Population:Sex	112.875	0.0113
Abdomen mass ~ 1 ± Population	113.001	0.0106
Abdomen mass ~ 1 ± Season + Population	114.792	0.0043
Abdomen mass ~ 1 ± Season + Sex + Population ± Sex:Season + Population:Season	115.030	0.0038
Abdomen mass ~ 1 ± Sex + Population	115.072	0.0038
Abdomen mass ~ 1 ± Season + Sex + Population	117.354	0.0012
Abdomen mass ~ 1 ± Sex + Population ± Population:Sex	117.901	0.0009
Abdomen mass ~ 1 ± Season + Sex + Population ± Sex:Season + Population:Season + Population:Sex	118.007	0.0009
Abdomen mass ~ 1 ± Season + Sex + Population ± Population:Sex	119.971	0.0003
Abdomen mass ~ 1 ± Season + Population ± Population:Season	119.976	0.0003
Abdomen mass ~ 1 ± Season + Sex + Population ± Population:Season	122.519	0.0001
Abdomen mass ~ 1 ± Season + Sex + Population ± Population:Season + Population:Sex	125.180	0.0000
**Thorax:Body mass ratio candidate models**		
Thorax:Body ~1 ± Season + Sex + Population ± Sex:Season + Population:Sex	−487.649	0.6113
Thorax:Body ~1 ± Season + Sex + Population ± Sex:Season	−485.922	0.2578
Thorax:Body ~1 ± Season + Sex + Population ± Sex:Season + Population:Season + Population:Sex	−482.772	0.0534
Thorax:Body ~1 ± Season + Sex + Population ± Sex:Season + Population:Season	−481.361	0.0263
Thorax:Body ~1 ± Sex + Population ± Population:Sex	−481.047	0.0225
Thorax:Body ~1 ± Season + Sex + Sex:Season	−480.441	0.0166
Thorax:Body ~1 ± Sex + Population	−479.008	0.0081
Thorax:Body ~1 ± Season + Sex + Population ± Population:Sex	−476.415	0.0022
Thorax:Body ~1 ± Season + Sex + Population	−474.484	0.0008
Thorax:Body ~1 ± Sex	−473.543	0.0005
Thorax:Body ~1 ± Season + Sex + Population ± Population:Season + Population:Sex	−471.307	0.0002
Thorax:Body ~1 ± Season + Sex + Population ± Population:Season	−469.580	0.0001
Thorax:Body ~1 ± Season + Sex	−469.184	0.0001
Thorax:Body ~1 ± Population	−461.033	0.0000
Thorax:Body ~1 ± Season + Population	−455.948	0.0000
Thorax:Body ~1	−454.852	0.0000
Thorax:Body ~1 ± Season + Population ± Population:Season	−450.882	0.0000
Thorax:Body ~1 ± Season	−449.842	0.0000
**Thorax mass candidate models**		
Thorax mass ~ 1 ± Season + Sex + Population	−1204.551	0.7300
Thorax mass ~ 1 ± Season + Sex + Population ± Population:Sex	−1199.945	0.0730
Thorax mass ~ 1 ± Season + Sex + Population ± Population:Season	−1199.904	0.0715
Thorax mass ~ 1 ± Season + Sex + Population ± Sex:Season	−1199.461	0.0573
Thorax mass ~ 1 ± Sex + Population	−1198.101	0.0290
Thorax mass ~ 1 ± Season + Sex	−1195.801	0.0092
Thorax mass ~ 1 ± Season + Sex + Population ± Population:Season + Population:Sex	−1195.203	0.0068
Thorax mass ~ 1 ± Season + Sex + Population ± Sex:Season + Population:Sex	−1194.878	0.0058
Thorax mass ~ 1 ± Season + Population	−1194.865	0.0058
Thorax mass ~ 1 ± Season + Sex + Population ± Sex:Season + Population:Season	−1194.788	0.0055
Thorax mass ~ 1 ± Sex + Population ± Population:Sex	−1193.626	0.0031
Thorax mass ~ 1 ± Population	−1191.250	0.0009
Thorax mass ~ 1 ± Season + Sex + Sex:Season	−1190.640	0.0007
Thorax mass ~ 1 ± Season + Sex + Population ± Sex:Season + Population:Season + Population:Sex	−1190.107	0.0005
Thorax mass ~ 1 ± Season + Population ± Population:Season	−1190.027	0.0005
Thorax mass ~ 1 ± Sex	−1189.298	0.0004
Thorax mass ~ 1 ± Season	−1185.265	0.0000
Thorax mass ~ 1	−1181.654	0.0000
**Forewing area candidate models**		
Forewing area ~ 1 ± Season + Population ± Population:Season	472.549	0.3379
Forewing area ~ 1 ± Season + Population	473.279	0.2346
Forewing area ~ 1 ± Season + Sex + Population	473.646	0.1952
Forewing area ~ 1 ± Season + Sex + Population ± Population:Season	473.800	0.1808
Forewing area ~ 1 ± Season + Sex + Population ± Population:Sex	478.988	0.0135
Forewing area ~ 1 ± Season + Sex + Population ± Sex:Season	479.092	0.0128
Forewing area ~ 1 ± Season + Sex + Population ± Population:Season + Population:Sex	479.262	0.0118
Forewing area ~ 1 ± Season + Sex + Population ± Sex:Season + Population:Season	479.267	0.0117
Forewing area ~ 1 ± Season + Sex + Population ± Sex:Season + Population:Sex	484.423	0.0009
Forewing area ~ 1 ± Season + Sex + Population ± Sex:Season + Population:Season + Population:Sex	484.729	0.0008
Forewing area ~ 1 ± Population	494.484	0.0000
Forewing area ~ 1 ± Sex + Population	497.180	0.0000
Forewing area ~ 1 ± Sex + Population ± Population:Sex	502.619	0.0000
Forewing area ~ 1 ± Season	558.223	0.0000
Forewing area ~ 1 ± Season + Sex	562.388	0.0000
Forewing area ~ 1	565.636	0.0000
Forewing area ~ 1 ± Season + Sex + Sex:Season	567.363	0.0000
Forewing area ~ 1 ± Sex	570.446	0.0000

*Note*: Best fit model for each trait is highlighted in red. Models were fit with Gaussian distribution and ranked by BIC score and model weight.

**TABLE 4:** Candidate generalized linear model for mature oocyte counts.

Mature oocyte count candidate models	BIC	Weight
Oocytes ~ 1 ± Population ± Season	1650.049	0.3694
Oocytes ~ 1 ± Population ± Season + Season:Population	1651.738	0.1588
Oocytes ~ 1 ± Population ± Season + Year + Year:Population	1651.963	0.1419
Oocytes ~ 1 ± Population ± Season + Year + Year:Population ± Year:Season	1652.033	0.1370
Oocytes ~ 1 ± Population ± Season + Year	1652.428	0.1124
Oocytes ~ 1 ± Population ± Season + Year + Season:Population	1655.801	0.0208
Oocytes ~ 1 ± Population ± Season + Year + Year:Season	1655.958	0.0193
Oocytes ~ 1 ± Population ± Season + Year + Season:Population ± Year:Population	1656.758	0.0129
Oocytes ~ 1 ± Season	1657.136	0.0107
Oocytes ~ 1 ± Population ± Season + Year + Season:Population ± Year:Population ± Year:Season	1657.145	0.0106
Oocytes ~ 1 ± Population ± Season + Year + Season:Population ± Year:Season	1658.811	0.0046
Oocytes ~ 1 ± Season + Year	1661.212	0.0014
Oocytes ~ 1 ± Season + Year + Year:Season	1666.230	0.0001
Oocytes ~ 1 ± Population ± Year + Year:Population	7214.004	0.0000
Oocytes ~ 1 ± Population ± Year	7230.434	0.0000
Oocytes ~ 1 ± Population	7236.518	0.0000
Oocytes ~ 1	7500.162	0.0000
Oocytes ~ 1 ± Year	7504.577	0.0000

*Note*: The best fit model is highlighted in red. Models were fit with negative binomial distribution and ranked by BIC score and model weight.

To quantify the association between each independent variable and dependent variable for our glms, we calculated the effect size with the statistic Eta squared (*η* ^2^) which is the ratio of each group's sum of squares to the total sum of squares. It is interpreted as the percentage of variance accounted for by each variable in the glm. For the negative binomial model, we relied on model coefficients to determine the predominant effect. We further performed the rank‐based nonparametric Kruskal–Wallis test to determine whether there were differences between groups and then a post hoc Dunn test (with a Bonferroni correction for multiple testing) to determine which groups were different. Circularity scores, aspect ratios, and mean shape distances were not normally distributed, and various transformations of the measurements did not yield normal distributions. In these cases, we relied on the rank‐based nonparametric Kruskal–Wallis test with post hoc Dunn test (with a Bonferroni correction) to identify differences between groups.

We used standardized major axis regression (SMA) implemented in the R package “smatr” (R Core Team, 2013; Warton et al., 2006) to test for the effects of rearing conditions and population on metabolic rate. SMA controls for the relationship between metabolic rate and mass (in our case, whole‐body wet mass) like an analysis of covariance, but it accounts for the fact that both metabolic rate and mass are measured with error. We used SMA to fit the metabolic scaling relationship between ln(VCO_2_) and ln(mass) for all individuals within particular combinations of the factors sex, population (NA and CR), and rearing season (summer and autumn). We first tested whether the scaling relationship between MR and mass was similar between the levels of our independent factors (i.e., testing for difference in slopes). When there was no evidence of a mass × factor interaction, we fit a common slope and then tested whether there was a significant effect of the factor on the elevation of the relationship between MR and mass (i.e., a difference in the mass‐specific metabolic rate) or a significant shift along the x‐axis between factor levels (i.e., a difference in mass). Because females and males did not differ significantly in the scaling relationship between MR and mass or in mass‐specific MR (Table 5), the sexes were combined for all subsequent analyses. The scaling exponents relating ln(VCO_2_) and ln(mass) were >1. While this deviates from the broad interspecific pattern where metabolic rate scales with mass to the ¾ power, intraspecific scaling exponents frequently deviate from this expectation (Glazier, 2005; Greenlee et al., 2014). These scaling exponents may also have differed if we had chosen a different measure for mass, for example, with wings removed.

**TABLE 5:** Summary of a standardized major axis regression (sma) used to fit the metabolic scaling relations between ln (VCO2) and ln (mass) to test for effects of sex on metabolic rate (MR).

Season, trait	Population	Sex	H_0_: Equal slopes slope (95% CI)^1^	H_0_: no elevation difference Y‐intercept (95% CI)^2^
Summer, resting MR	N. American		LR = 0.006, *df* = 1, *p* = .93	Wald = 2.82, *df* = 1, *p* = .09
			Common slope: 3.17 (2.56, 3.92)	
		Male		0.75 (0.45, 1.06)
		Female		0.79 (0.50, 1.08)
	Costa Rica		LR = 0.64, *df* = 1, *p* = .42	Wald = 0.14, *df* = 1, *p* = .70
			Common Slope: 2.81 (2.30, 3.46)	
		Male		0.53 (0.28, 1.79)
		Female		0.55 (0.29, 0.81)
Autumn, resting MR	N. American		LR = 1.11, *df* = 1, *p* = .29	Wald = 0.11, *df* = 1, *p* = .73
			Common slope: 3.59 (2.80, 4.52)	
		Male		0.98 (0.60, 1.36)
		Female		1.01 (0.64, 1.37)
	Costa Rica		LR = 0.13, *df* = 1, *p* = .71	Wald = 0.23, *df* = 1, *p* = .63
			Common Slope: 2.79 (2.12, 3.67)	
		Male		0.80 (0.40, 1.20)
		Female		0.77 (0.36, 1.17)
Summer, max flight MR	N. America		LR = 0.17, *df* = 1, *p* = .67	Wald = 3.79, *df* = 1, *p* = .09
			Common slope: 2.06 (1.64, 2.58)	
		Male		1.90 (1.69, 2.12)
		Female		1.80 (1.61, 1.99)
	Costa Rica		LR = 0.64, *df* = 1, *p* = .42	Wald = 2.73, *df* = 1, *p* = .85
			Common slope: 2.31 (1.78, 2.99)	
		Male		2.03 (1.74, 2.32)
		Female		1.92 (1.65, 2.19)
Autumn, max flight MR	N. America		LR = 2.743, *df* = 1, *p* = .09)	Wald = 0.07, *df* = 1, *p* = .78
			Common slope: 1.90 (1.42, 2.58)	
		Male		1.84 (1.59, 2.09)
		Female		1.83 (1.59, 2.06)
	Costa Rica		LR = 0.03, *df* = 1, *p* = .84	Wald = 0.2.28, *df* = 1, *p* = .13
			Common slope: 3.00 (2.00, 4.47)	
		Male		2.50 (1.87, 3.13)
		Female		2.32 (1.67, 2.98)

We used mass‐corrected resting and flight MRs to test for the statistical significance of interactions between population and rearing condition on metabolic rates, which would indicate a difference in plasticity between NA and CR monarchs. Mass‐corrected MRs were obtained by taking the residual for each individual from the SMA fits of ln(VCO_2_) as a function of ln(mass) and adding back the average ln(VCO_2_) to obtain a meaningful scale as in Hoekstra et al. (2013). The mass‐corrected MRs were used as the dependent variable in linear models to test for the effects of population, rearing season, and the statistical interaction between population and rearing season.

## 3 RESULTS

### 3.1 Both NA and CR monarchs exhibit seasonal plasticity in female reproduction

**FIGURE 3:**
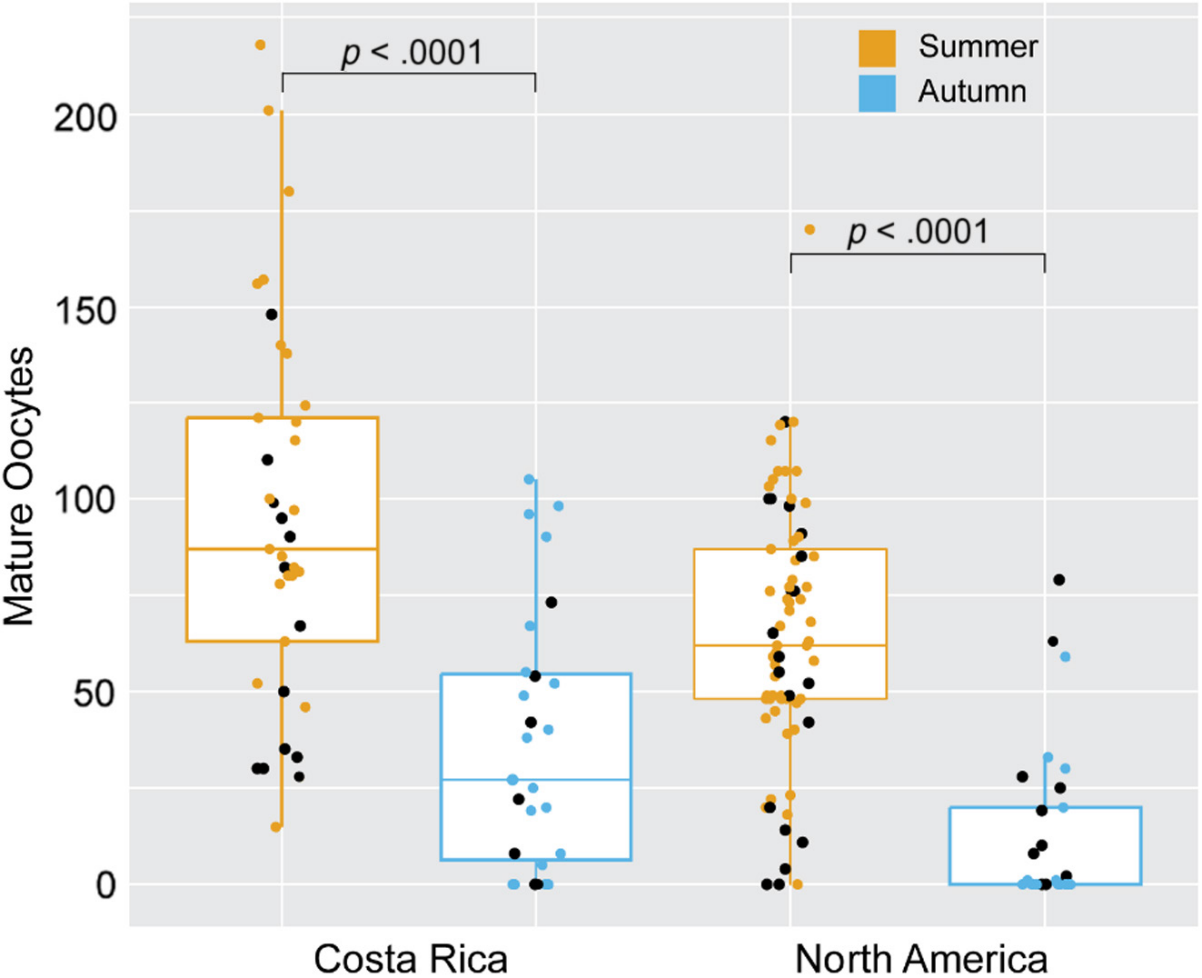
Boxplot of mature oocytes in female monarchs. Both CR and NA monarchs have decreased the numbers of mature oocytes in response to autumn rearing, relative to summer rearing. Significant differences between rearing seasons are indicated on the plot. Black dots highlight individuals also assayed for metabolic rates.

The number of mature oocytes present in a female monarch's abdomen was best explained by the additive effects of season and population (Table 6). Both CR and NA monarchs had fewer mature oocytes when reared in autumn (Figure 3, Kruskal–Wallis *χ* ^2^ = 64.32, *df* = 3, *p* = 7.02e−14), consistent with the known seasonal reproductive diapause. Season had a larger effect size estimate (1.31, 95% CI [0.94, 1.66]) than did population (−0.62, 95% CI [−0.97, −0.28]) with little CI overlap. Although our model suggests there was a significant effect of population, differences between CR and NA monarchs within each seasonal rearing environment were not significant after correcting for multiple tests.

**TABLE 6:** Summary of the best fit general linear model (glm) for abdomen mas, thorax mass, the ratio of thorax: body mass, and forewing area.

Independent variable	*df*	Coefficient	Sum sq	Mean sq	*F* value	*p* value	*η* ^2^
Abdomen mass, *R* ^2^ = 0.096							
Sex	1	−0.1988	0.31	0.3105	3.382	.06755.	0.017
Season	1	−0.0853	0.284	0.2843	3.097	.08015.	0.016
Sex:Season	1	0.3314	1.168	1.1684	12.728	.00046***	0.064
Thorax mass, *R* ^2^ = 0.189							
Season	1	0.0045	0.00078	0.000775	10.39	.0015**	0.047
Sex	1	0.0050	0.00129	0.001294	17.36	4.81E−05***	0.078
Population	1	0.0048	0.00106	0.001058	14.19	.00022***	0.064
Thorax: body ratio, *R* ^2^ = 0.274							
Season	1	0.0446	0.001	0.00095	0.272	.60253	0.001
Sex	1	0.0966	0.1072	0.10724	30.591	1.12E−07***	0.125
Population	1	0.0534	0.0417	0.04172	11.9	.0007***	0.049
Season:Sex	1	−0.0742	0.0614	0.06138	17.51	4.48E−05***	0.071
Sex:Population	1	−0.0459	0.024	0.02399	6.844	.00966**	0.028
Forewing area, *R* ^2^ = 0.37							
Season	1	0.2003	7.62	7.62	19.564	1.49E−05***	0.053
Population	1	0.6713	43.29	43.29	111.09	<2e−16***	0.3
Season:Population	1	0.4099	2.41	2.41	6.173	.0137*	0.017

*Note*: Each model's *R* ^2^ is reported along with the significance and effect size of each independent variable in the model. *η* ^2^ (Eta squared) is a measure of effect size that can be interpreted as the amount of variance accounted for by each variable in the best fit glm. *p < .05; **p < .01; ***p < .001.

### 3.2 Components of body mass differ in seasonal plasticity between sexes and populations

Total body mass (the combined mass of the thorax and abdomen) did not differ significantly between populations, rearing seasons, or sexes (mean = 0.0845 grams, Kruskal–Wallis *χ* ^2^ = 11.4, *df* = 7, *p* = .12). However, abdomen mass was seasonally plastic in both NA and CR monarchs, and this plasticity differed between the sexes (Table 6). A model including season, sex, and their interaction explained ~10% of variation in abdomen mass (*R* ^2^ = 0.096; Table 6). Males reared in summer had lighter abdomens than females reared in summer (female mean = 0.0352 g vs male mean = 0.043 g, Kruskal–Wallis *χ* ^2^ = 16.8, *df* = 7, *p* = .019, Dunn test with Bonferroni correction, *p* = .0035), and males increased abdomen mass in response to autumn (autumn mean = 0.0454 g vs summer mean = 0.0352 g, Dunn test with Bonferroni correction, *p* = .0043).

**FIGURE 4:**
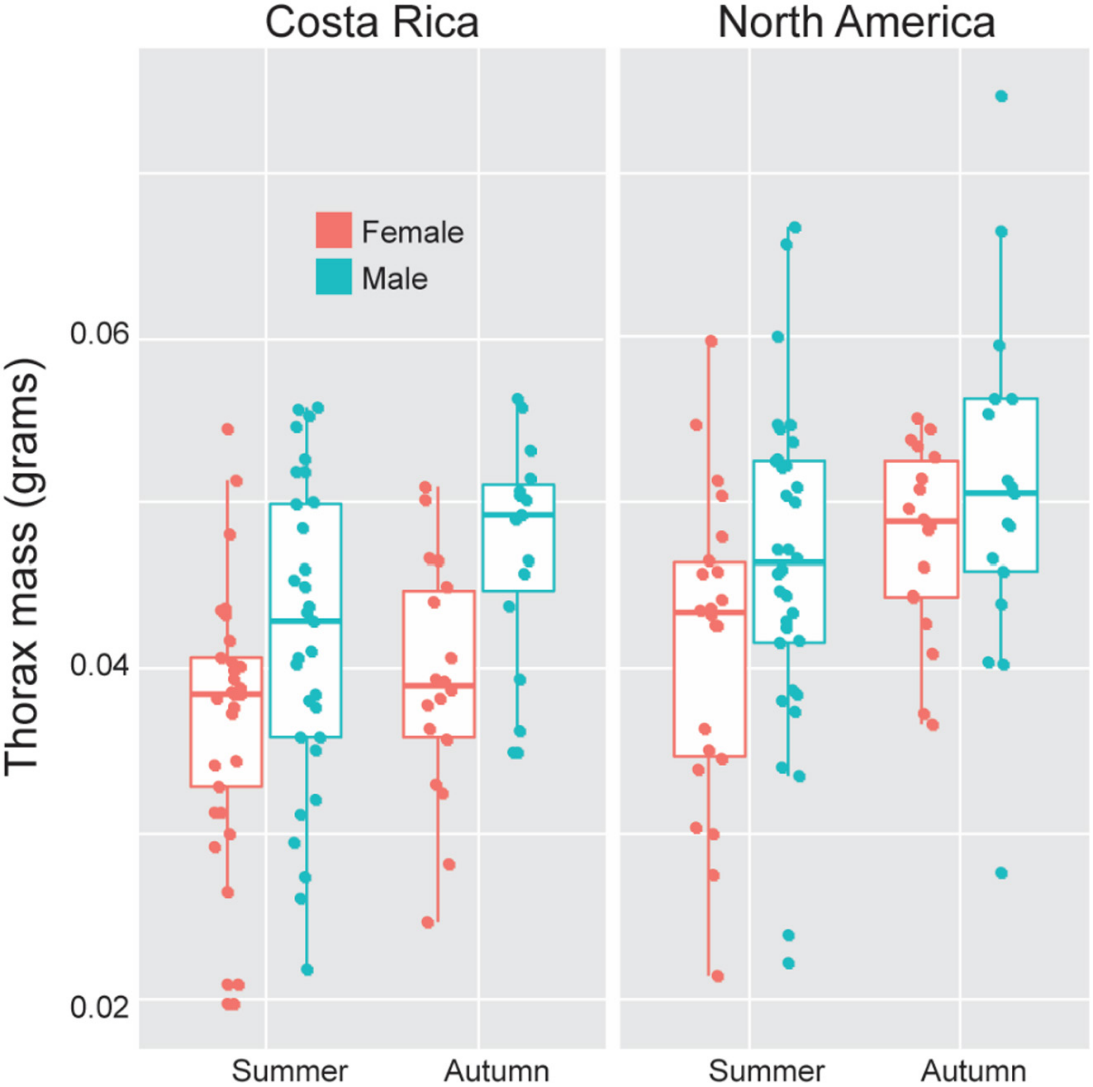
Boxplot of thorax mass measured in grams.

Thorax mass was seasonally plastic in both populations, with no evidence for a statistical interaction between season and population (Table 6). A model including rearing season, sex, and population explained 19% (*R* ^2^ = 0.19) of the variation in thorax mass (Table 6). An individual's thorax was likely to be heavier if population was NA, sex was male, and season of development was autumn (Figure 4). A post hoc test found no significant differences between NA males and females or CR males and females reared in either season, though the difference between CR males and females was nearly significant in autumn (Kruskal–Wallis *χ* ^2^ = 38.67, *df* = 7, *p* < .0001, Dunn test with Bonferroni correction, NA: *p* = .88, *p* = 1.0, CR: *p* = .48, *p* = .08, summer and autumn, respectively).

**FIGURE 5:**
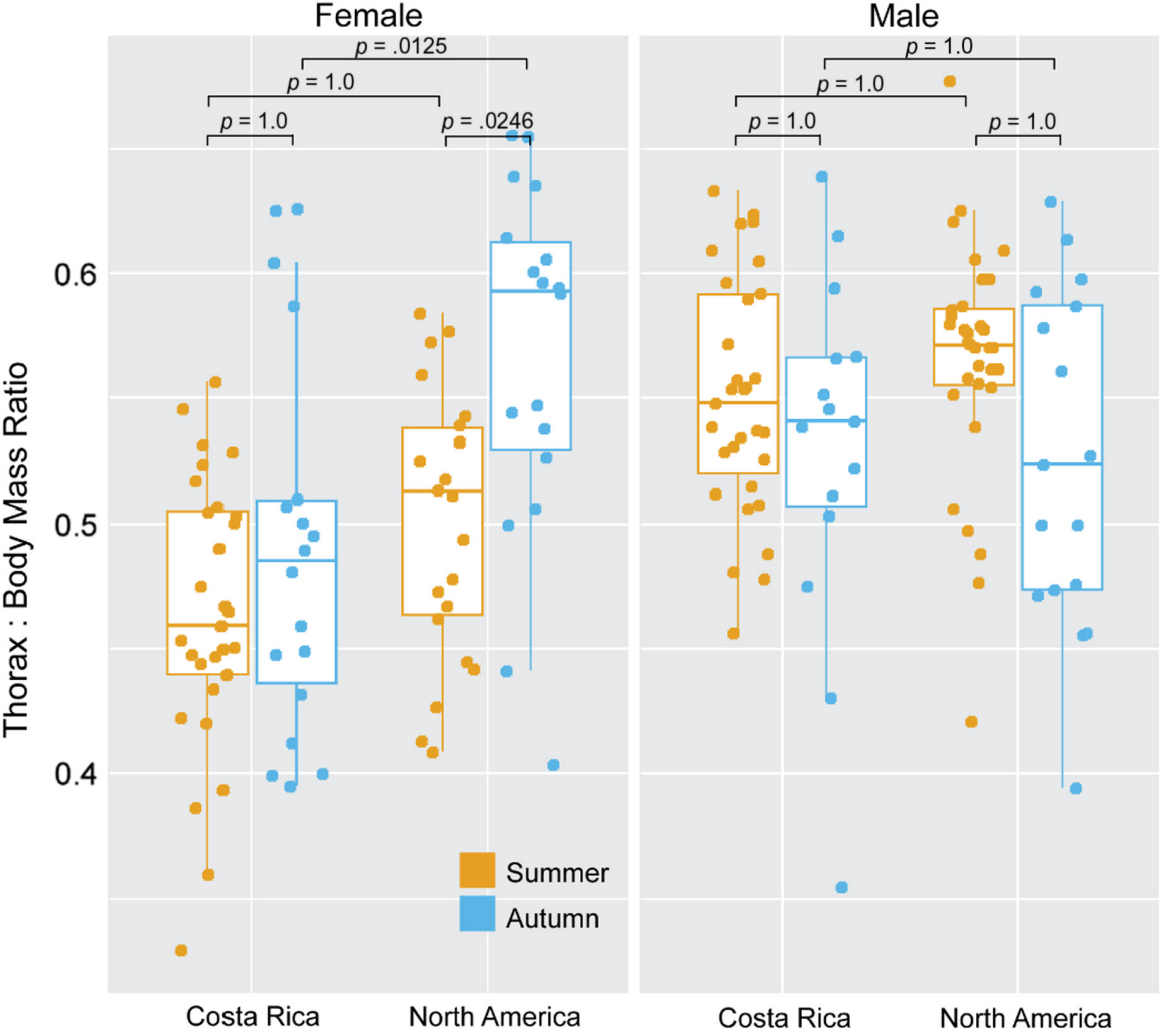
Boxplot of the ratio of thorax mass to total body (thorax + abdomen) mass. Scores above 0.5 indicate that an individual has invested more of their total mass in the thorax than in the abdomen. NA female monarchs increase investment in thorax tissue in autumn. *p*‐values for differences between season, sex, and population are indicated on the plot.

The ratio of thorax mass to total body mass was seasonally plastic in females, particularly in NA monarchs (Table 6). A model including population and sex, as well as interactions between population and sex and between sex and season, explained 27% of the variation in the thorax: body mass ratio (*R* ^2^ = 0.27). Sex had the largest effect with males having higher thorax: body mass ratios than females. NA monarchs had higher thorax: body mass ratios than did CR monarchs. Females increased the thorax: body mass ratio when reared in autumn relative to summer, and this effect of season was significant in NA but not in CR females (Figure 5, Kruskal–Wallis *χ* ^2^ = 52.97, *df* = 7, *p* < .0001, Dunn test with Bonferroni correction, CR: *p* = 1 and NA: *p* = .0246). Autumn‐reared NA female thorax: body mass ratios were significantly greater than those of autumn‐reared CR females (Figure 5, Dunn test with Bonferroni correction *p* = .0125). In summary, investment in thorax mass as a fraction of total body mass exhibits a sex‐specific plasticity that was significant in NA monarchs, with NA females developing a more male‐like pattern of investment in autumn versus summer.

### 3.3 Only NA monarchs exhibit seasonal plasticity in wing size

**FIGURE 6:**
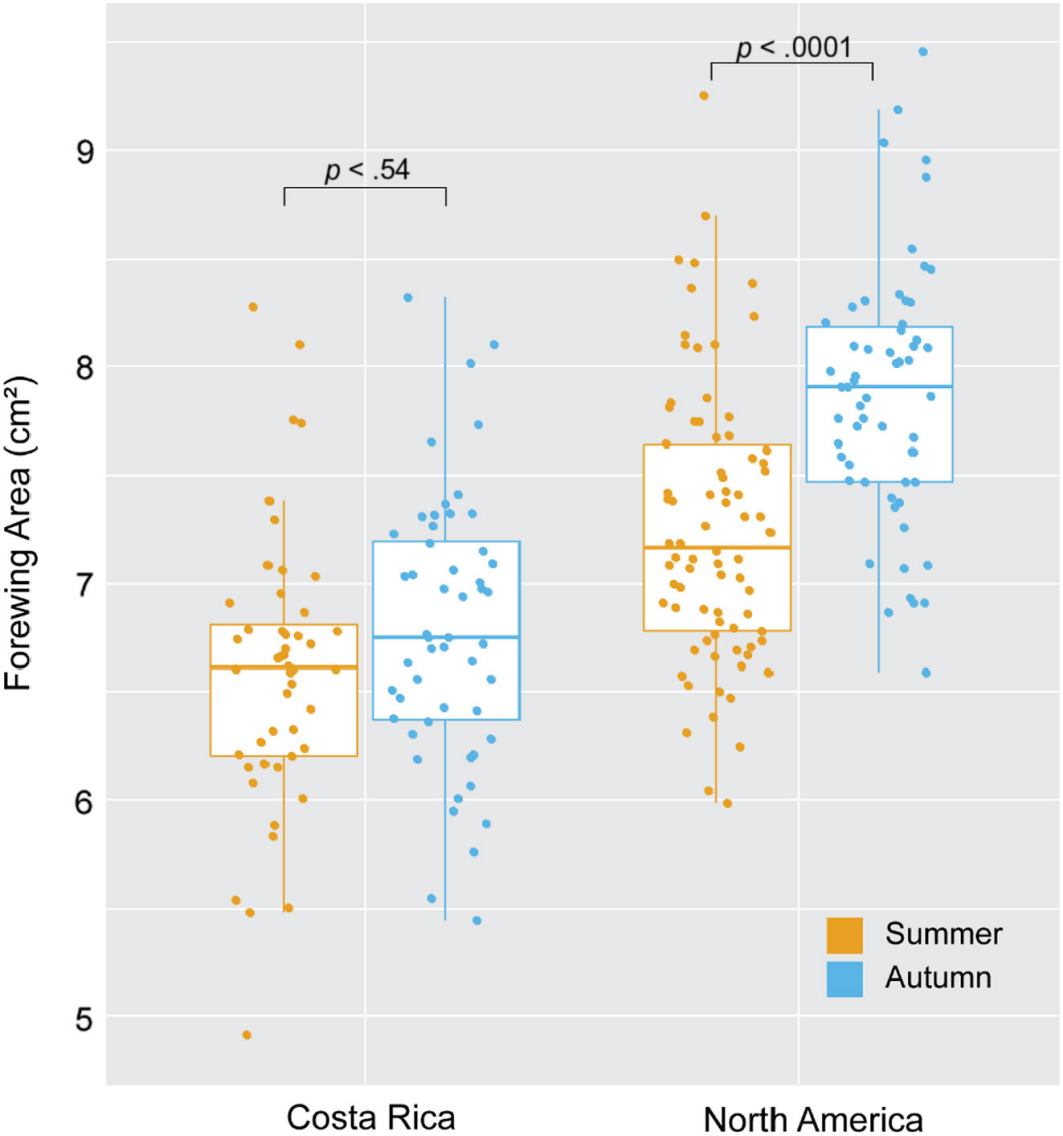
Boxplot of the forewing area measured in cm^2^. NA monarchs reared in autumn had significantly larger forewings relative to those reared summer, while this difference was reduced and not significant in CR monarchs. *p*‐values for differences between seasons in each population are indicated on the plot.

Wing area was seasonally plastic in NA monarchs. Variation in wing area was best explained by a model that included the effects of rearing season and population, as well as their interaction (*R* ^2^ = 0.37; Table 6). NA monarchs reared in autumn had on average 8% larger forewings than the NA summer‐reared monarchs (Figure 6, summer mean = 7.26 cm^2^ vs autumn mean = 7.87 cm^2^, Kruskal–Wallis *χ* ^2^ = 90.68, *df* = 3, *p* < .0001, Dunn test with Bonferroni correction, *p* < .0001) and 16% larger forewings than the CR autumn‐reared monarchs. CR monarch forewing area was not significantly different between summer‐ and autumn‐reared monarchs (Figure 6, summer mean = 6.56 cm^2^ vs autumn mean = 6.79 cm^2^, Dunn test with Bonferroni correction, *p* = .54).

### 3.4 Neither NA nor CR monarchs exhibit seasonal plasticity in wing shape

**FIGURE 7:**
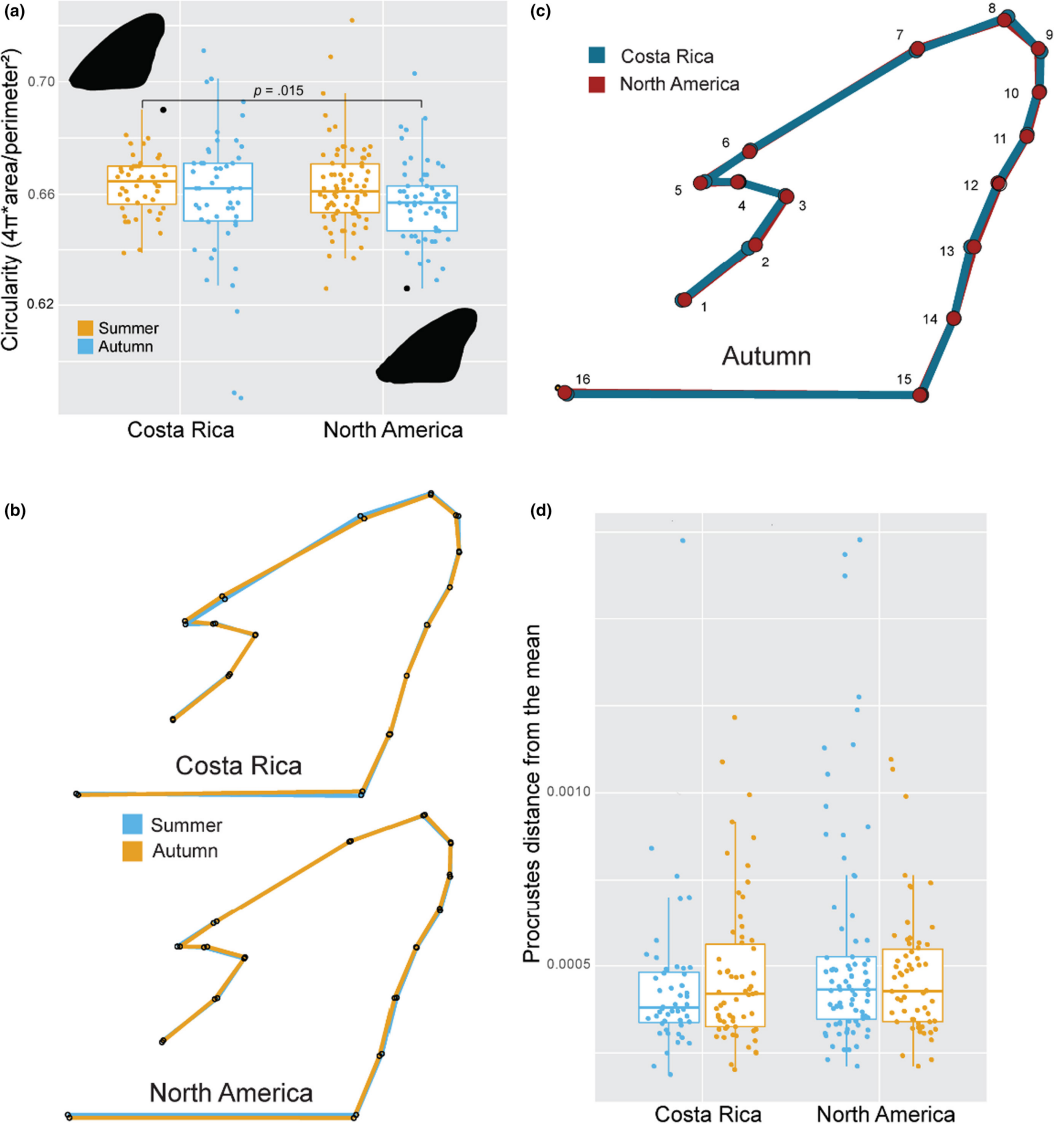
Wing shape measures. (a) Boxplot of forewing circularity scores. Lower circularity scores indicate an individual with a more elongated wing. CR monarchs reared in summer had higher circularity scores than NA monarchs reared in autumn. The *p*‐value of the only significant difference between groups is indicated on the plot. Examples of both a less elongated CR monarch wing and a more elongated NA monarch wing are highlighted in black. (b) Mean shape of the autumn forewings (yellow) plotted on top of the mean summer (blue) forewings by population. Each dot is the consensus mean coordinate for landmarks 1–16. Straight lines are drawn between landmarks to outline the forewing. (c) Comparison of mean forewing shape of Costa Rican (blue) and North American (red) monarchs reared outdoors in autumn. Each dot is the consensus mean coordinate for landmarks 1–16. Straight lines are drawn between landmarks to outline the forewing. (d) A boxplot of Procrustes distances from the mean consensus forewing shape. Each dot represents the cumulative distance of 16 coordinates (landmarks) from their respective mean shape coordinate. There were no significant differences between groups.

In contrast, measures of forewing shape did not differ between NA and CR monarchs and showed little to no difference between seasons. Variation in forewing aspect ratio was not explained by sex, season, population, or any of their interactions (Kruskal–Wallis *χ* ^2^ = 7.37, *df* = 7, *p* = .39). Circularity of the forewing, where a value of 1 is a perfect circle and decreasing scores indicate more polygonal (angular) forewings, was not seasonally plastic in either population, although NA monarchs trended toward more angular wings in autumn (Figure 7a, Kruskal–Wallis *χ* ^2^ = 8.72, *df* = 3, *p* = .03, Dunn test with Bonferroni correction, NA: *p* = .085, and CR: *p* = .85). Geometric morphometric analysis did not reveal any differences in mean shape between NA and CR forewings in either season (Figure 7b), and neither population exhibited significant difference between seasons in this measure of forewing shape (Figure 7c). To quantify variability in forewing shape, we measured the distance of each individual forewing's landmark to the respective consensus mean landmark and summed those distances. Total distance from the mean shape did not vary by population or season (Figure 7d, Kruskal–Wallis *χ* ^2^ = 3.58, *df* = 3, *p* = .31).

### 3.5 Metabolic rates were seasonally plastic in CR but not NA monarchs

**FIGURE 8:**
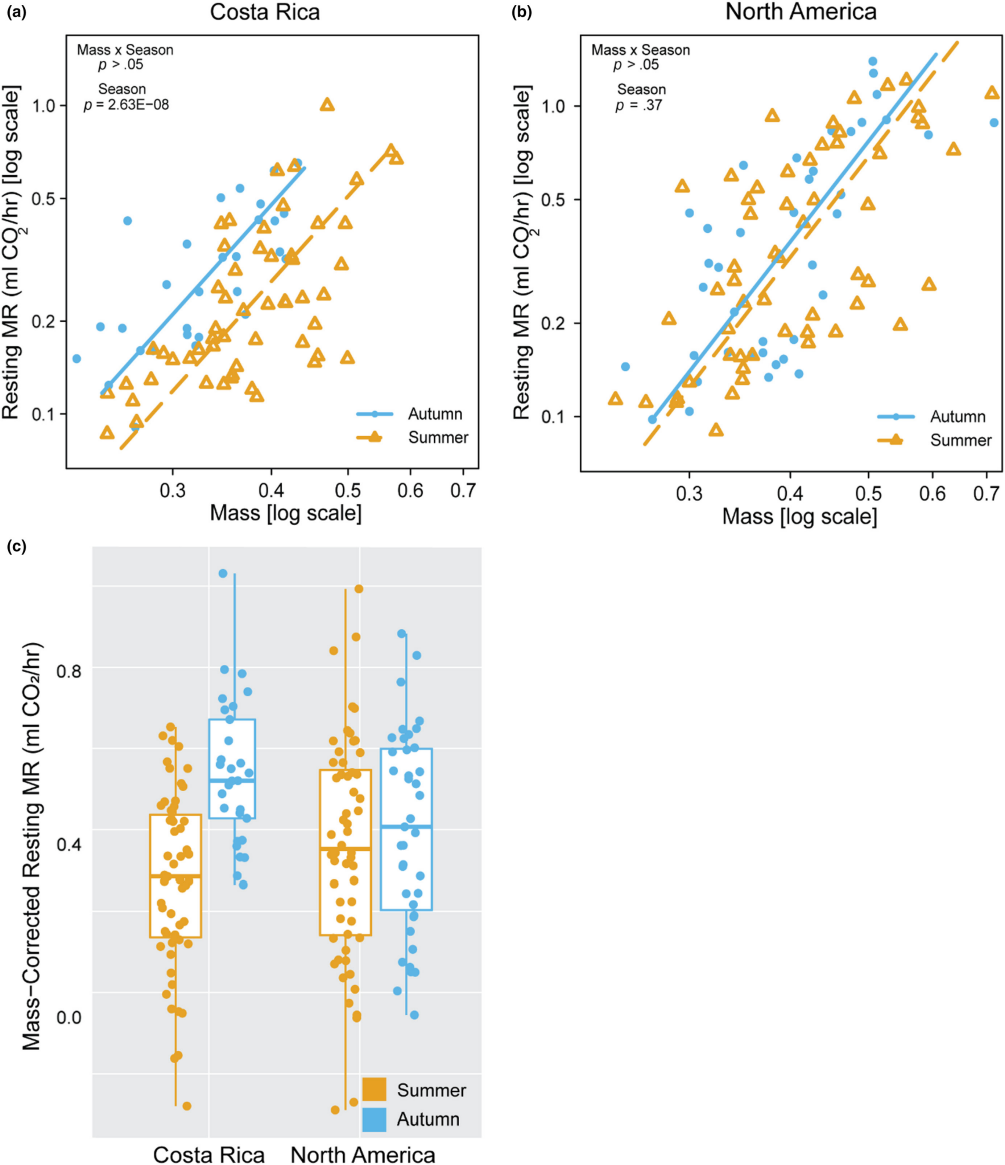
Effects of population and seasonal rearing conditions on resting metabolic rates (MR). (a) Resting MR were significantly increased in CR monarchs reared in autumn relative to summer, (b) while rearing season did not affect the resting MR of NA monarchs. (c) There was a significant effect of the interaction between population and rearing season on mass‐corrected resting MR (population × rearing, *p* = .004), with the maintenance of similar resting metabolic rates across seasonal rearing environments in NA but not in CR monarchs. Male and female data are plotted together, as the sexes did not differ in patterns of MR (Table 5).

Resting MR of NA monarchs did not differ significantly between seasons (mass × season, *p* = .55; season, *p* = .37) (Figure 8b and Table 7). However, autumn‐reared CR monarchs had significantly higher resting MR relative to summer‐reared CR monarchs (mass × season, *p* = .86; season, *p* = 2.63E−08) (Figure 8a and Table 7). Variation in mass‐corrected MR was explained by a significant interaction between rearing season and population (*p* = .004; Table 8), with only CR monarchs exhibiting a difference in resting MR between seasons (Figure 8c). Resting MR of summer‐reared NA and CR monarchs was not significantly different, but resting MR of CR monarchs was significantly greater than that of NA monarchs reared in autumn (Table 9).

**TABLE 7:** Summary of a standardized major axis regression (sma) used to fit the metabolic scaling relations between ln (VCO2) and ln (mass), and to test for effects of rearing season on metabolic rate (MR) within populations.

Population, trait	Season	H_0_: Equal slopes slope (95% CI)^a^	H_0_: no elevation difference Y‐intercept^b^
Costa Rica, resting MR		LR = 0.03, *df* = 1, *p* = .86 Common slope: 2.84 (2.41, 3.34)	Wald = 30.96, *df* = 1, *p* = 2.63E−08
	Autumn		0.81* (0.41, 1.16)
	Summer		0.56* (0.31, 0.83)
N. America, resting MR		LR = 0.35, *df* = 1, *p* = .55 Common slope: 3.34 (2.86, 3.89)	Wald = 0.78, *df* = 1, *p* = .37
	Autumn		0.89 (0.66, 1.12)
	Summer		0.84 (0.62, 1.06)
Costa Rica, max flight MR		LR = 1.79, *df* = 1, *p* = .19 Common slope: 2.20 (1.85, 2.61)	Wald = 4.13, *df* = 1, *p* = .04
	Autumn		2.25* (1.95, 2.55)
	Summer		2.12* (1.87, 2.37)
N. America, max flight MR		LR = 0.00, *df* = 1, *p* = .97 Common slope: 2.03 (1.70, 2.42)	Wald = 1.69, *df* = 1, *p* = .19
	Autumn		1.89 (1.73, 2.05)
	Summer		1.84 (1.69, 1.99)

*Note*: *p < .05; **p < .01; ***p < .001.

^a^ Test of common slope from a Type II regression model.

^b^ Significant differences in y‐intercept within a common slope are evidence of differences in MR across the range of masses measured.

**TABLE 8:** Summary of general linear model used to test for effects of population and rearing season on mass‐corrected MR.

Trait	Independent variable	*df*	Sum sq	Mean sq	*F*‐value	*p*‐value
Mass‐corrected resting MR	Population	1	0.001	0.0014	0.03	.8746
	Season	1	0.879	0.8789	15.64	.0001***
	Population:Season	1	0.490	0.4900	8.72	.0036**
Mass‐corrected flight MR	Population	1	0.036	0.0360	0.70	.404
	Season	1	0.252	0.2515	4.89	.0283*
	Population:Season	1	0.023	0.0232	0.45	.5026

*Note*: *p < .05; **p < .01; ***p < .001.

**TABLE 9:** Summary of a standardized major axis regression (sma) used to fit the metabolic scaling relations between ln (VCO2) and ln (mass) and to test for the effects of population on metabolic rate (MR) within seasons.

Season, trait	Population	H_0_: Equal slopes slope (95% CI)^a^	H_0_: no elevation difference Y‐intercept^b^
Summer, resting MR		LR = 0.53, *df* = 1, *p* = .46 Common slope: 3.03 (2.62, 3.51)	Wald = 2.82, *df* = 1, *p* = .09
	N. America		0.72 (0.53, 0.91)
	Costa Rica		0.64 (0.44,0.84)
Autumn, resting MR		LR = 1.68, *df* = 1, *p* = .19 Common slope: 3.18 (2.67, 3.81)	Wald = 6.597, *df* = 1, *p* = .01)
	N. America		0.82* (0.62, 1.31)
	Costa Rica		0.97* (0.41, 1.16)
Summer, max flight MR		LR = 1.01, *df* = 1, *p* = .31 Common slope: 2.20 (1.85, 2.61)	Wald = 0.10, *df* = 1, *p* = .74
	N. America		1.91 (1.75, 2.06)
	Costa Rica		1.92 (1.75, 2.09)
Autumn, max flight MR		LR = 4.09, *df* = 1, *p* = .04	
	N. America	2.02 (1.53, 2.67)	
	Costa Rica	3.28 (2.24, 4.79)	

*Note*: *p < .05; **p < .01; ***p < .001.

^a^ Test of common slope from a type II regression model.

^b^ Significant differences in y‐intercept within a common slope are evidence of differences in MR across the range of masses measured.

**FIGURE 9:**
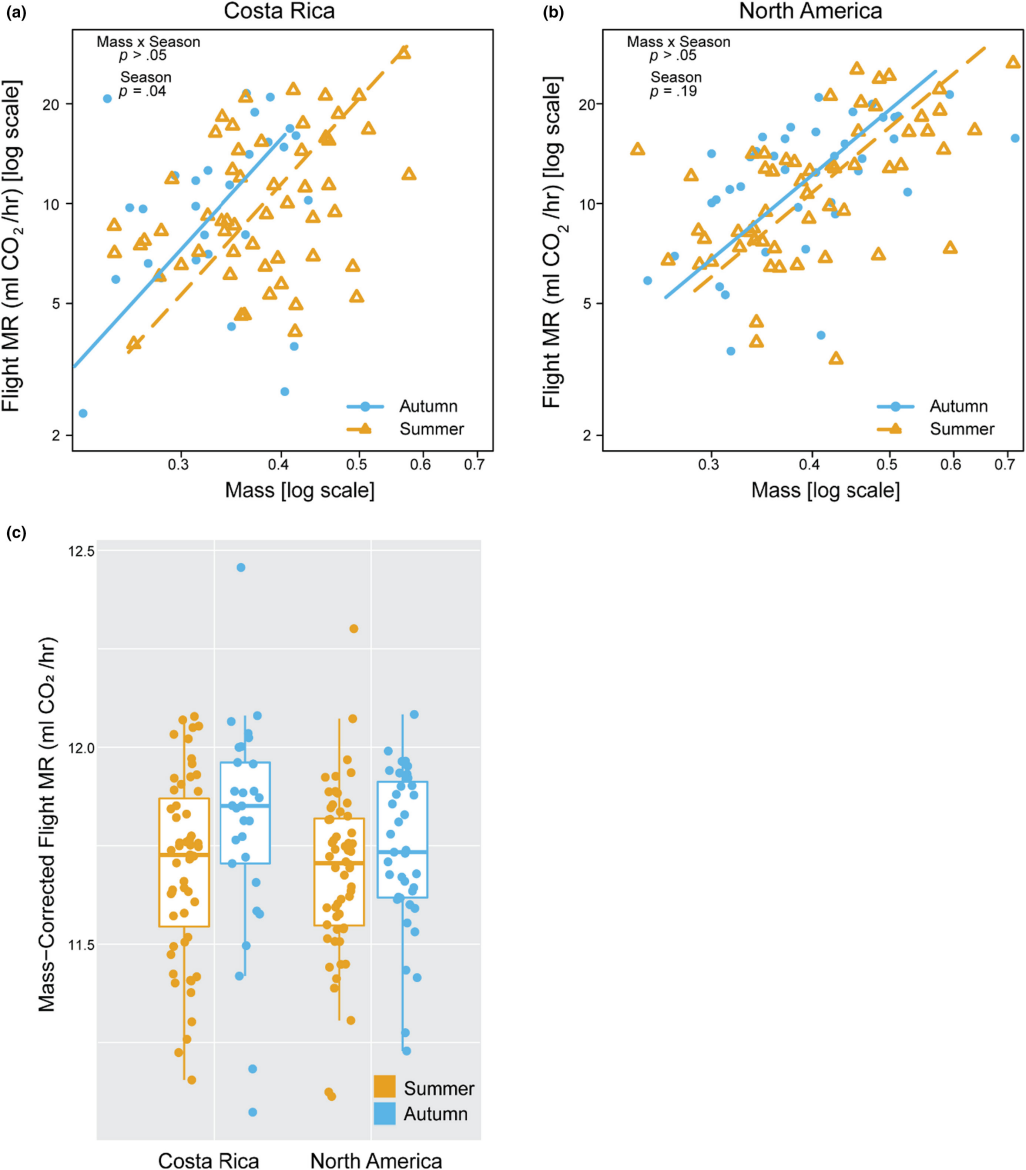
Effects of population and seasonal rearing conditions on flight metabolic rates (MR). (a) CR monarchs had significantly greater flight MR when reared in autumn relative to summer. (b) Flight MR were slightly elevated in, but not significantly different between autumn and summer‐reared NA monarchs. (c) Mass‐corrected flight MR showed a similar pattern, with increased flight MR in autumn‐relative to summer‐reared monarchs (rearing, *p* = .03) and a larger magnitude of difference in CR monarchs. However, there was no statistically significant effect of the interaction (population × rearing, *p* = .50). Male and female data are plotted together, as the sexes did not differ in patterns of metabolic rate (Table 5).

Similar to patterns for resting MR, CR monarchs had elevated maximal flight MR when reared in autumn relative to summer (mass × season, *p* = .19, season *p* = .04) (Figure 9a and Table 7), while maximal flight MR in NA monarchs did not differ between seasons (mass × season, *p* = .97; season, *p* = .19) (Figure 9b and Table 7). When we corrected maximal flight MR for mass, there was a significant effect of season (*p* = .0283; Table 8) but no significant interaction between rearing season and population (Table 8). However, the difference in mass‐corrected flight MR between seasons appeared larger in CR relative to NA monarchs (Figure 9c). Flight MR of summer‐reared NA and CR monarchs were not significantly different, but autumn‐reared CR and NA monarchs differed in the scaling relationship with mass, with larger NA monarchs maintaining lower flight MR than CR monarchs (Table 9).

To test whether monarchs from different populations or reared in different seasons differed in their ability to recover their resting MR after flight, we calculated the percent recovery of RMR (post‐flight RMR/pre‐flight RMR × 100) for each individual. Monarchs from both populations reared under both seasons had significantly elevated post‐flight resting MR relative to pre‐flight resting MR (average percent recovery = 118.62%; paired *t*‐test including all individuals *t* = −3.2759, *p* = .001). However, this pattern was similar among populations and rearing conditions, and there were no significant differences between pre‐ and post‐flight RMR within any of the population‐by‐rearing condition groups, likely a function of the reduced power to detect this small difference in these smaller subsets of the data.

## 4 DISCUSSION

We compared ancestral temperate (NA) and derived tropical (CR) monarch populations for the extent of seasonal plasticity in physiological and morphological traits suspected to be adaptive for monarch migration and overwintering. We predicted that plasticity would be lost in monarch populations that have dispersed into more stable, tropical habitats, such as Costa Rica. We found that the non‐migratory CR descendants of the migratory NA population retain some, but not all ancestral seasonal trait plasticity. This suggests that seasonal plasticity in monarchs can be lost in a piecemeal fashion in the absence of selective pressures for its maintenance. The maintenance of metabolic rates in autumn compared to summer, plus the increase in wing size and thorax mass relative to total body mass in NA monarchs, suggests that these traits may be important for migration success and that the regulation of these traits may be critical to maintaining alternative summer and autumn phenotypes.

Mass differences in the abdomen and thorax are consistent with different selective pressures facing females versus males as well as NA versus CR populations. Autumn rearing induces an apparent shift in resources in females presumably from egg mass to flight muscle, consistent with the idea that successful autumn migration is critical for both sexes. While NA male and female monarchs were not significantly different in thorax mass in either season in our comparisons, a previous experiment that compared thorax mass in NA monarchs found significant differences between thorax mass in males and females (Davis & Holden, 2015). Particularly in summer, we saw a similar trend toward larger male thoraxes, and sex was a significant predictor of thorax mass in our glm. Though not to the same degree as NA females, CR females also responded to autumn by increasing the thorax to body mass ratio though the difference comes from a decrease in abdomen mass rather than an increase in thorax mass in autumn. In summary, CR females retained seasonally plastic reproduction, but the seasonal shift in allocation to thorax mass may be eroding. Further investigation of plasticity in resource allocation into reproductive and flight muscle tissues are warranted, as well as investigation of whether other abiotic factors (e.g., drought or host‐plant quality) may induce reproductive diapause and maintain plasticity for this trait in tropical monarch populations.

Forewing size was the most divergent morphological trait between NA and CR monarchs. Consistent with other work comparing migratory and resident monarch populations, we found CR monarchs had smaller wings than NA monarchs (Altizer & Davis, 2010; Beall & Williams, 1945; Dockx, 2007; Freedman et al., 2020; Li et al., 2016). However, unlike previous work, our study explicitly compared monarchs reared in the NA monarch's migratory range in summer and autumn. We found that forewing size was seasonally plastic in NA but not in CR monarchs. Previous measurements from a study of museum specimens collected in North America between 1878 and 2017 noted that autumn‐collected individuals had larger wings than summer (Freedman & Dingle, 2018). Our data suggest that this difference is at least partly explained by seasonal plasticity in wing size rather than differential mortality during migration (Davis et al., 2020; Flockhart et al., 2017). The smaller forewing size of CR and other resident monarch populations plus the CR monarchs' lack of plasticity suggests that adaptation to the local environment post‐dispersal may have selected for smaller wing size. Meanwhile, large wing size is likely under constant selection in migratory NA monarch populations during autumn, as large wing size is associated with longer flight in butterflies (Altizer & Davis, 2010; Flockhart et al., 2017; Li et al., 2016). Thus, this might be an example where seasonal heterogeneity maintains plasticity in wing size in NA monarchs, with the summer‐like small wing trait fixed in resident monarch populations that experience more summer‐like conditions throughout the year. Investigating the flight and fitness consequences of these changes in wing morphology would be particularly useful for assessing whether this is an example of the loss of plasticity through adaptive assimilation.

The importance of wing shape to migration is less clear. Previous work found differences in shape between some resident and migratory monarchs (Altizer & Davis, 2010; Dockx, 2007; Freedman et al., 2020; Satterfield & Davis, 2014), while other population comparisons did not find differences (Freedman et al., 2020; Li et al., 2016). Between our three measures of wing shape (geometric morphometrics, aspect ratio, and circularity), the only significant shape difference was in forewing circularity between autumn‐reared NA monarchs and summer‐reared CR monarchs, but the difference was small and the distributions were largely overlapping. We suggest that the difference seen in circularity when comparing wild‐caught CR monarchs to NA monarchs (Altizer & Davis, 2010) could be driven by developmental environment rather than population. However, we found no evidence of seasonal plasticity in wing shape in either population, consistent with findings from Flockhart et al. (2017) which found no relationship between wing roundness or aspect ratio and distance flown in NA migrators. However, others have noted differences in aspect ratio when comparing wild‐caught to indoor‐reared NA monarchs (Davis et al., 2020) and when comparing NA individuals caught earlier in the migration season to individuals caught later (Satterfield & Davis, 2014).

In contrast to the prediction that NA monarchs relative to CR monarchs might exhibit greater plasticity in metabolic rates to support flight during migration, we observed that metabolic rates were affected by seasonal rearing only in CR monarchs. Autumn‐reared CR monarchs had elevated resting and flight metabolic rates relative to summer‐reared monarchs, while NA monarchs maintained similar and lower resting and flight metabolic rates across seasons. There are two, non‐mutually exclusive, ways to interpret this pattern. First, the NA population may have seasonal plasticity in underlying physiology that maintains similar metabolic rates across seasonal environments, with the plastic mechanisms that maintain metabolic rate across the seasons lost in the CR population. Second, if the CR population has lost either the maternal provisioning or developmental mechanisms appropriate for the shorter photoperiod days of autumn, then the elevated metabolic rates in autumn‐reared CR monarchs may be the consequence of coping with environmental stress during development. That stress, however, cannot be attributed to differences between reproductive output or host plant between the populations, as monarchs from both populations significantly decreased egg counts in response to autumn and consumed common milkweed in both summer and autumn in our common garden experiment. We noted that while common milkweed differs from CR's native tropical milkweed host (*Asclepias curassavica*), this did not result in differences in metabolic rate between the populations in the summer, suggesting that any effect of host plant on population differences in metabolic rate in our study must interact with the effect of seasonal rearing.

Our results were similar to previous studies of metabolic rates in NA and CR monarchs that were reared in summer (Zhan et al., 2014), although that study used somewhat different measures of metabolic rates and did detect differences in flight metabolic rate between migratory NA and resident Florida monarchs. Zhan et al. (2014) also found evidence for positive selection and divergent expression of collagen IV alpha‐1 and alpha‐2 in adult thoracic muscle tissue between migratory and non‐migratory populations of monarchs. These proteins are essential for muscle morphogenesis and function (Schnorrer et al., 2010) and have been interpreted as evidence for the evolution of flight efficiency in migrating monarchs (Zhan et al., 2014). Flight is energetically demanding, and selection for long‐distance migratory flight may favor more efficient flight relative to shorter duration flight (Rankin & Burchsted, 1992). Our results lend support to this hypothesis, as we found that NA monarchs maintained similar resting and flight metabolic rates across seasons. We suggest that migration is supported not by increased metabolic output but likely through other seasonally plastic changes (e.g., in wing area, as we observed, and/or muscle structures) that enable more efficient flight. These results contrast with some other migratory and dispersing insects that have higher metabolic rates compared to their non‐migratory and non‐dispersing counterparts (Crnokrak & Roff, 2002; Niitepõld et al., 2009; Tanaka & Okuda, 1996; Zera et al., 1997). Of these examples, NA monarchs migrate the farthest and live the longest. Thus, the maintenance of low metabolic rates may enable monarchs to better survive the months‐long overwintering period in Mexico where they consume very little food. Our observation that NA butterflies are able to maintain low flight MR unlike CR butterflies reared in autumn may also indicate that NA monarch physiology enables more efficient flight in the presence of accumulated lipid reserves during migration (Brower et al., 2006; Gibo & McCurdy, 1993; Schroeder et al., 2020).

## AUTHOR CONTRIBUTIONS

**Ayse Tenger‐Trolander:** Conceptualization (equal); data curation (equal); formal analysis (equal); funding acquisition (equal); investigation (equal); methodology (equal); project administration (equal); visualization (equal); writing – original draft (equal); writing – review and editing (equal). **Cole R Julick:** Conceptualization (equal); data curation (equal); formal analysis (equal); funding acquisition (equal); investigation (equal); methodology (equal); project administration (equal); visualization (equal); writing – original draft (equal); writing – review and editing (equal). **Wei Lu:** Data curation (supporting); formal analysis (supporting); investigation (supporting); methodology (supporting); visualization (supporting); writing – original draft (supporting); writing – review and editing (equal). **D. Andre Green II:** Conceptualization (supporting); funding acquisition (equal); investigation (supporting); writing – original draft (supporting); writing – review and editing (supporting). **Kristi L Montooth:** Conceptualization (equal); formal analysis (equal); funding acquisition (equal); investigation (equal); methodology (equal); project administration (equal); resources (equal); supervision (equal); writing – original draft (equal); writing – review and editing (equal). **Marcus Kronforst:** Conceptualization (equal); funding acquisition (equal); investigation (equal); project administration (equal); resources (equal); supervision (equal); writing – original draft (equal); writing – review and editing (equal).

## FUNDING INFORMATION

This work was supported by the Graduate Research Fellowship Program, the National Institute of Health Genetics and Regulation Training Grant (T32 GM07197), the US Fish and Wildlife Service (Award F17AC01222), the National Science Foundation Grants (IOS‐1452648 and IOS‐1736249), and the National Institutes of Health Grant (GM131828).

## CONFLICT OF INTEREST STATEMENT

The authors declare no conflicts of interest.

## Supporting information


Data S1.

## Data Availability

All data are included in Data S1 Data.xlsx.
